# Is the femoral intramedullary alignment already actual in total knee arthroplasty?

**DOI:** 10.1186/s40634-022-00563-y

**Published:** 2023-02-14

**Authors:** Andrea Tecame, Andrea Gambineri, Pierangelo Za, Paolo Adravanti

**Affiliations:** 1 Department of Orthopaedic and Trauma Surgery, Città di Parma Clinic, Piazzale Athos Maestri 5, Parma, Italy; 2 Department of Orthopaedic and Trauma Surgery, Campus Bio-Medico University of Rome, Via Alvaro del Portillo 200, Rome, Italy

**Keywords:** Intramedullary, Extramedullary, Femoral cut, Alignment, TKA

## Abstract

Clinical outcomes and overall alignment after total knee arthroplasty (TKA) depend on femoral component positioning in the sagittal and the coronal plane, making choice of the distal femoral cutting guide crucial. Currently, there is no consensus on the potential advantage of an extramedullary (EM) guide compared to an intramedullary (IM) guide in TKA. The IM guide is the most widely used system for making the distal femoral cut although evidence for its superiority over the EM guide is lacking. However, inaccuracies arising with the IM guide include location of the rod entry point in the coronal plane, femoral canal diameter, femoral bowing, and structural features of the rod. Furthermore, the invasive procedure is associated with increased risk of postoperative blood loss, thromboembolic complications, and intraoperative fractures. While the EM guide has no such difficulties, its accuracy depends on the instruments used. Studies have reported results not inferior to the IM guide and a lower number of postoperative complications. Patient-specific instrumentation (PSI) and robotic and computer-assisted TKA have achieved excellent clinical and radiographic results and can overcome the problems inherent to the IM and the EM guide. Authors performed a systematic review of the literature and proposed a narrative review to summarize the characteristics of the IM and the EM guide and compare the advantages and disadvantages of each, as well as their limitations in comparison with new technologies. Authors also expressed their expert opinion.

## Introduction

Two types of alignment guides for achieving accurate bone cuts are the intramedullary (IM) and the extramedullary (EM) guide. The femoral IM guide is widely applied in TKA owing to its ease of use. While studies have shown that a more accurate femoral bone cut may be obtained with the IM guide [7, 10, 11, 14], these studies are dated with limitations regarding inadequate technique and preoperative radiographic templating [1, 28, 59] and difficult-to-identify intraoperative landmarks with the EM guide [7, 11, 60]. Current studies have shown that IM guides don’t always ensure the expected component placement [33, 34]. Many factors can affect IM alignment accuracy, including location of the rod entry point in the coronal plane, femoral canal diameter, femoral bowing, and structural features of the rod [5, 16, 41, 52]. In addition, the invasive procedure is associated with increased risk of postoperative blood loss, thromboembolic complications, and intraoperative fractures [20, 21, 43]. Finally, patient-specific instruments (PSI) and robotic-assisted systems that provide highly accurate alignment are coming into wider use [35] though still not feasible in many settings [49]. For this narrative review we summarize recent evidence on two alignment systems for distal femoral bone cuts in TKA and compare the IM and EM guide with the most recent technologies. Despite the narrative nature of this review, the authors performed a systematic review of the literature. Two independent reviewers (P.Z. and A.T.) searched PubMed, EMBASE, and the Cochrane Library up to May 31, 2022. The electronic search strategy used was alignment AND (intramedullary OR extramedullary guide). In cases of disagreement, a third reviewer (P.A) made the final decision. The inclusion criteria were full-text basic science studies that compared the intramedullary with the extramedullary alignment and studies that analyze the main characteristics of one or both methods . Humans and cadaveric studies were included. Studies not written in English were not considered.

## Intramedullary guide

The IM guide is the most widely used system for distal femoral cut in TKA despite lack of clear evidence for its superiority over an EM guide. The entry point for the femoral IM rod is a crucial factor for femoral component positioning and restoring the mechanical axis in TKA since it affects the extension gap and limb alignment in both the coronal (varus/valgus) and the sagittal (flexion/extension) plane [30, 48]. Even minor deviations in the insertion point can result in misalignment by several degrees [24, 46]. Errors can result from non-ideal entry point location, improper IM rod length, rod diameter, and rotation of the IM rod [46]. Identification of the ideal femoral entry point has been elusive probably due to high inter-individual anatomical variability, making it difficult to determine an entry point that fits all patients. Reed and Gollish [52] reported that the ideal entry point should be about 6.6 mm medial to the center of the notch, on average. Novotny et al. [46] suggested that the optimal entry point for the IM rod should be determined as a ratio. Wangroongsub and Cherdtaweesup [62] concluded that the entry point should be 1.5 ±2.01 mm medial and 12 ±2.72 mm superior to the top of the femoral intercondylar notch. In their 3D analysis of the femoral entry point, Jianlin Xiao et al. [64] argued that using a point 2.94 ±1.12 mm (range 0.79–4.91 mm) medial and 6.01 ±2.09 mm (range 2.49–9.51 mm) anterior to the deepest point of the intercondylar notch yields an error margin of less than 2°. Errors can also arise from incorrect intramedullary rod thickness and length: a rod that is too long can trigger insertion errors, leading to femoral bowing or other deformities of the distal femur, while a rod with a small radius may not achieve adequate fit within the canal. Nufio-Siebrecht [47] underscored that error can be minimized by increasing IM rod diameter and length. Novotny [46] recommended use of a rod 9 mm in diameter and 228.6 mm (9 inches ) in length with a long guiding rod to minimize chance of error. They argued that rotation can decrease valgus and impart flexion/extension to the anteroposterior cut. The effect for small degrees of malrotation is negligible: for each 10° of malrotation the effect is ~1° in the sagittal plane and ~0.3° in the frontal plane [46]. G. Maderbacher et al. [33] simulated by means of computer-aided engineering software the distal femoral cut using intramedullary alignment rods with the rod gradually aligned from 40° of external to 40° of internal rotation. They found that rotational changes of the distal femoral alignment guides affect both the coronal and the sagittal cutting plane. But because femoral bowing is very frequent in Asian populations, caution is warranted when evaluating data obtained from such populations. Furthermore, the negative results of the IM technique obtained in Asian patient samples should not be extended to Caucasian patient samples, in which lower limbs conformation is different. In their level II study, G. Maderbacher et al. [34] used the IM rod in 40 knees for distal femoral cut in TKA and found that only 25% (10/40) of the gamma angle measurements were within a range between 0° and 3° of flexion, which is the theoretical optimal range of flexion of the femoral component. They concluded that the appropriate sagittal femoral component alignment cannot be ensured with the use of IM alignment rods.

## Extramedullary guide

Early studies from the late 1980s and early 1990s on the EM guide reported inferior results compared to the IM guide in which the anterosuperior iliac spine was used as an intraoperative reference for the EM guide [6, 7, 11]. More recent studies with modern EM guidance systems have obtained better results. Baldini and Adravanti [1] developed a set of EM instruments calibrated with preoperative templating to locate the mechanical axis and perform the distal femoral cut without violating the femoral canal. They found that the EM technique was more accurate than the IM technique in the sagittal plane. But because their study included only varus osteoarthritic knees, the conclusions may not be extended to all types of deformity. Matsumoto et al. [37] used a similar EM system and reported femoral component coronal alignment within 0° ±3° of the mechanical axis in 98% of patients. In their level I study, Woon-hwa Jung et al. [19] used an EM instrument to measure interfemoral head center distance for distal femoral bone cut in TKA and compared the results with data from a second group in which an IM guide was used. The overall lower limb alignment was within 0 ±3° of the mechanical axis in all EM group patients. The gamma angle was 2.5 ±1.0° in the IM and 2.3 ±1.7° in the EM group, but no statistically significant differences between the EM and the IM group were found in the coronal and the sagittal alignment of the femoral component. The EM guide is free from complications related to the violation of the femoral canal but much of its precision depends on the characteristics of the instruments. Several authors reported non-inferiority of the extramedullary guide compared to the intramedullary guide with a lower complication rate. The EM guide offers an excellent alternative when use of an IM rod is difficult or impossible. Studies [1, 4] have suggested using the EM guide for bilateral, simultaneous TKA, major femoral extraarticular deformities, ipsilateral long-stemmed hip arthroplasty, and when hardware (distal femoral plates and screws, IM nails) is present.

## Intramedullary versus extramedullary guides

There is no consensus on whether the EM femoral guide is superior to the IM guide in TKA. One of the most invasive parts of TKA is violation of the IM femoral canal after use of IM instruments. Application of an IM guide for the femur can produce a variety of complications including blood loss, postoperative hypoxia, intraoperative fractures, and fat embolism [21, 27, 29]. In contrast, the EM method is associated with less morbidity and blood loss because the bone marrow is not invaded. However, identifying the anterosuperior iliac spine as an intraoperative landmark to locate the femoral head center for the EM guide is difficult because of the large soft tissue cover, tourniquets, and the anatomical variability of the proximal femur. In their metanalysis, Q. Tang et al. [58] found no statistically significant difference between the EM- and the IM-guided femoral cut for lower limb coronal alignment, coronal alignment of the femoral component, sagittal alignment of the femoral component, and operating time, but blood loss was less with use of the EM guide. Baldini and Adravanti [1] found that femoral component sagittal alignment within 0 ±2° (gamma angle) was achieved more often in the EM than in the IM group (90% versus 78% respectively). In their study investigating the influence of the IM and the EM guide on distal femoral cut in 6726 TKAs, Meding et al. [40] found no statistical difference in postoperative clinical scores or in survivorship at 15 years of follow-up; however, mean tibiofemoral (overall) anatomical alignment was statistically more accurate in the IM group.

## General concept of alignment

Proper prosthetic placement and overall limb alignment are crucial for successful TKA implant survivorship and prevention of early failure [12, 53, 56]. Among different types of alignment described in the literature [25], there is broad consensus that coronal and sagittal component mechanical alignment should be within ±3° of neutral alignment to best ensure implant survival [12, 34, 53, 54, 56]. However, not all studies supported a neutral mechanical axis as gold standard [32]. Parrate [50] performed a retrospective study on 398 TKAs, he considered as outliers 106 knees with post-operative mechanical alignment outside 0 ° ± 3 ° and found no difference in survivorship beetween 106 outliers and 292 weel-aligned knees at 15 years. Other authors [39, 61] reported a non-inferiority of clinical results if the pre-operative deformity is preserved or partially preserved, without however reaching the range -3 / +3°. In addition, the current expansion of the kinematic alignment, and the excellent outcomes reported so far, further cast doubt on the need to achieve an alignment within -3 / + 3 ° from the mechanical plane as a gold standard. Consistent with the new concepts of alignment on the coronal and the sagittal plane, use of an EM guide correlates much closer with the concepts of knee kinematics and better individualized alignment. When using the IM guide on the coronal plane, the degree of valgus is limited to what that the guide shows (generally 0 to 9°). Differently, taking into account condylar wear and observing the rule of re-establishing the knee’s pre-arthritic condition, the EM guide overcomes these limitations and constraints. In addition, the sagittal plane maintains its anatomy by leaning on the anterior cortex and thus follows femur anatomy.

Practicing a robotic assisted knee prosthetic replacement allows to bypass the problem of femoral alignment on the coronal plane, therefore of the use of a intra or extra medullary guide, since the distal femoral cut is carried out according to a preoperative computed tomography scan of the knee and consequently on a three-dimensional planning. There is no need to violate the medullary canal or to use further anatomical landmarks. However, the robotic assisted method remains highly expensive and not accessible to all surgeons. This makes the dilemma of which technique is best to optimize the distal femoral cut still current.

Emily L. Hampp, et al. [15] compared the precision and accuracy of bone cuts and positioning of components on 6 cadavers (12 knees), six knees performed with standard technique and 6 knees with robot assisted technique, demonstrating greater accuracy and precision of the robot-assisted technique compared to the manual one, especially for the management of the varus-valgus alignment on the coronal plane. The error on the distal femoral cut showed a median of 0.5 ° and 2.6 ° respectively for the assisted and manual robotic cut, without a substantial difference in the management of flexion-extension.

James D. Sires et al. [57] performed 45 TKA knee arthroplasty and analysed bone cuts, made using the MAKO Total Knee system (Stryker) and saw that for the distal femoral cut the maximum error was 1.20mm, 1.00mm, 1.80mm with an average error of 0.32mm , 0.28mm, 0.46mm for depth, valgus and flexion respectively. This precision allowed them to obtain an overall alignment within 3 ° of the preoperative planning in 100% of cases. KA also depends on the anatomical axis and therefore on the correct positioning of the intramedullary landmark. The advantage of the kinematic technique lies in being able to predict the thickness of the resections, the measurement of the thickness of the bone cuts allowing to perform an important check and consequently to carry out the necessary corrections or recut. This technique allows the patient's knee to be brought back to a pre-osteoarthritis condition without affecting the soft tissues. It is known that KA reduces the recovery time, improves functional outcomes, reduces residual symptoms after TKA, increases patient satisfaction with good survivorship at a short term follow-up. Howell, Shelton and Hull [18] have done a 10-year follow-up of 191 cases with a survival rate of 98.8%.

The robot-assisted technique allows to pursue a kinematic or mechanical alignment, being able to refer with extreme precision to the anatomical axes. In a recent systematic review and meta-analysis [26] the robot-assisted knee arthroplasty showed more accurate component positioning and placement within target zones, however, this procedure is more expensive and requires more operating time and there is no clear superiority over the conventional technique in terms of clinical scores, satisfaction and implant survivorship. The literature is limited with regard to the accuracy of bone cuts with robotic system.

In these terms we can say that the robotic technique is highly accurate and can satisfy both kinematic and mechanical alignment with extreme precision. However, it is not yet clear whether this advantage can justify the increase in economic expenditure.

## New trends

Patient-specific instrumentation (PSI), robotic and computer-assisted TKA were introduced with the aim to improve alignment without violating the femoral canal. PSI entails the use of customized cutting blocks generated from a pre-operative three-dimensional model created using computed tomography (CT) or magnetic resonance imaging (MRI). The aim is to facilitate prosthesis implant by analysis of 3-D preoperative planning of TKA and simulation of the bone cuts. During this phase coronal orientation and depth of bone resection are planned. Customized cutting guides that fit on the patient’s anatomy are then manufactured. However, there is no consensus regarding the accuracy and the reliability of PSI and many studies have reported controversial results. It cannot be considered a gold standard in TKA, and so cannot be recommended for routine use. No substantial improvement in component alignment, surgical time, blood loss or functional outcomes has been reported. Mattei et al. [38] suggested the use of PSI in particular situations such as post-traumatic osteoarthritis with severe deformities (e.g., femoral or tibial fractures healed with malalignment), in which preoperative planning may be difficult and use of an IM rod difficult or impossible. Computer-assisted surgery (CAS) for TKA seems to provide more precise component placement in coronal, sagittal and rotational alignment, more accurate bone cuts, and better restoration of coronal limb alignment [55, 56]. Several meta-analysis studies [13, 17, 36, 51] found that CAS improves mechanical axis and implant survivorship but no clinical evidence of improvement in functional outcome. In a meta-analysis of 29 studies comparing CAS to conventional technique, Mason et al. [36] reported that femoral varus/valgus alignment was within 2° of the femoral mechanical axis in 90.4% of the CAS group patients versus 65.9% in the conventional group. Nam et al. [44] used a portable accelerometer-based surgical navigation system to record the hip center of rotation and femoral mechanical axis for the distal femoral cut in 48 patients undergoing TKA. They reported that 95.8% of the femoral components were placed within 90° ±2° of the femoral mechanical axis in the coronal plane and that overall lower extremity alignment was within 3° of neutral to the mechanical axis in 93.8% of cases. In their retrospective study, Biazzo A. et al. [3] compared the radiological results obtained from three groups of patients undergoing TKA via three different methods (*n*=97 patients available for radiological assessment). At a medium follow-up of 15 years, there was no statistically difference in the mean hip-knee-ankle (HKA) angle and the mean frontal femoral component angle (FFC) between the patients who underwent TKA via a computer-based alignment system and those in which a total IM alignment system and a total EM alignment system were used. Importantly, however, they did not consider the sagittal plane. In their systematic overview of meta-analyses, Kort et al. [26] reported that robot-assisted knee arthroplasty allows for more accurate component positioning and placement within target zones, but the results were inconclusive about whether it improves clinical scores, patient satisfaction or reduces complications and revision rates. In their review of clinical and radiological results of the MAKO CT-based robotic-assisted system for TKA, Batailler et al. [2] found lower postoperative pain scores, shorter time to hospital discharge, better functional scores, and better implant positioning with CT-based robotic-assisted TKA than with conventional TKA. Other studies reported similar functional results for both surgical techniques at 6 months and 1 year of follow up [23, 45]. Robotic-assisted TKA seems to be less invasive than conventional TKA, thus allowing better soft tissue protection and exposure, without robot-related complications. Several studies reported an increase in infection rate probably due to longer operating times at the beginning of the learning curve and fracture at the pin insertion site or pin breakage [31, 63]. Kayani et al. [22] found that seven robotic cases are necessary to gain an improvement in the operating time of robotic arm-assisted TKA and of surgical team stress levels without any learning curve effect on accuracy of implant position and limb alignment. Cool et al., Cotter et al., and Mont et al. reported that 90-day episode-of-care (EOC) costs were lower after robotic-assisted TKA [8, 9, 42].

## Conclusions

There is no clear superiority of one guide over another (IM vs EM). The choice of either alignment system should be determined by the patient's anatomy and the surgeon’s confidence and experience. Orthopedic societies should devote more effort to the development of more precise EM guides. In our opinion, surgeons not familiar with navigation systems or robotic-assisted TKA should consider using the EM guide for the distal femoral cut. This technique has been proven safe, fast, and accurate for reproducing a cut in both the coronal and the sagittal planes. Moreover, overall results are not inferior to the IM guide with fewer post-operative side effects. Use of robot-assisted systems and navigation obviate the distinction between the EM and the IM guide. Numerous studies have reported better results without added complications and with lower 90-day EOC costs.

## Abbreviations

TKA: Total knee arthroplasty
EM: Extramedullary
IM: Intramedullary
PSI: Patient-specific instrumentation
CT: Computed tomography
MRI: Magnetic resonance imaging
